# Study protocol for a randomized controlled trial of supportive parents – coping kids (SPARCK)—a transdiagnostic and personalized parent training intervention to prevent childhood mental health problems

**DOI:** 10.1186/s40359-024-01765-y

**Published:** 2024-05-13

**Authors:** T. Tømmerås, A. Backer-Grøndahl, A. Høstmælingen, H. Laland, M. B. Gomez, A. Apeland, L. R. A. Karlsson, A. A. Grønlie, S. Torsvik, G. E. Bringedal, Advisory Consortium, Aas Monica, Phillip Andrew Fisher, Frances Gardner, John Kjøbli, Ira Malmberg-Heimonen, Helene Amundsen Nissen-Lie

**Affiliations:** 1 Norwegian Center for Child Behavioral Development, Oslo, Norway; 2 Social, Genetic and Developmental Psychiatry Centre, Psychology and Neuroscience, Kings College, London, UK; 3 Stanford University, Graduate School of Education, Stanford, US; 4 Department of Social Policy and Intervention, University of Oxford, Oxford, UK; 5 Centre for Child and Adolescent Mental Health, Eastern and Southern Norway, Oslo, Norway; 6 Oslo Metropolitan University, Faculty of Social Work, Oslo, Norway; 7 University of Oslo, Department of Psychology, Oslo, Norway

**Keywords:** Randomized effectiveness trial, Parent-training intervention, Child mental health problems, Transdiagnostic prevention

## Abstract

**Background:**

To meet the scientific and political call for effective prevention of child and youth mental health problems and associated long-term consequences, we have co-created, tested, and optimized a transdiagnostic preventive parent-training intervention, Supportive parents – coping kids (SPARCK), together with and for the municipal preventive frontline services. The target group of SPARCK is parents of children between 4 and 12 years who display symptoms of anxiety, depression, and/or behavioral problems, that is, indicated prevention. The intervention consists of components from various empirically supported interventions representing different theorical models on parent–child interactions and child behavior and psychopathology (i.e., behavioral management interventions, attachment theory, emotion socialization theory, cognitive-behavioral therapy, and family accommodation intervention). The content and target strategies of SPARCK are tailored to the needs of the families and children, and the manual suggests how the target strategies may be personalized and combined throughout the maximum 12 sessions of the intervention. The aim of this project is to investigate the effectiveness of SPARCK on child symptoms, parenting practices, and parent and child stress hormone levels, in addition to later use of specialized services compared with usual care (UC; eg. active comparison group).

**Methods:**

We describe a randomized controlled effectiveness trial in the frontline services of child welfare, health, school health and school psychological counselling services in 24 Norwegian municipalities. It is a two-armed parallel group randomized controlled effectiveness and superiority trial with 252 families randomly allocated to SPARCK or UC. Assessment of key variables will be conducted at pre-, post-, and six-month follow-up.

**Discussion:**

The current study will contribute with knowledge on potential effects of a preventive transdiagnostic parent-training intervention when compared with UC. Our primary objective is to innovate frontline services with a usable, flexible, and effective intervention for prevention of childhood mental health problems to promote equity in access to care for families and children across a heterogeneous service landscape characterized by variations in available resources, personnel, and end user symptomatology.

**Trial registration:**

ClinicalTrials.gov ID: NTCT05800522

**Supplementary Information:**

The online version contains supplementary material available at 10.1186/s40359-024-01765-y.

## Introduction

Fifteen to 20 percent of Norwegian children aged three to 18 years have experienced mental health-related difficulties, and seven percent exhibit symptoms compatible with a mental health diagnosis [1]. Furthermore, children often have complex problems, and their symptoms often extend beyond a singular mental health domain or diagnosis [2, 3]. In other words, comorbidity within and across symptom domains is common. These children are at risk of negative developmental trajectories, characterized by poor mental and physical well-being, school absenteeism and dropout, reduced quality of life, and social and professional exclusion later in life [4–6]. To prevent negative life-course trajectories, there has been a societal and political emphasis in Norway on prevention and early intervention for vulnerable children and families [7]. However, the quality of frontline services for children and families varies and has been identified as one of the principal challenges for the municipal sector in Norway [8]. In this protocol, we describe a randomized controlled effectiveness study of a transdiagnostic parent training intervention in municipal frontline services to prevent child and youth mental health problems.

Over the past several decades, evidence-based interventions (EBI) have been developed and tested to prevent and treat mental health problems in youth populations [9]. However, the effectiveness of such interventions has remained stagnant or even declined [10, 11], and the extensive development and testing of interventions has not been reflected in the implementation and utilization of such programs [12]. Many children and families in need of effective treatment or preventive help, do not have access to it [13, 14]. One explanation that has been proposed for this lack of effectiveness, is that resource intensive EBIs targeting singular problem domains do not fit the comorbid problems of clients and the complex realities faced by professionals in frontline services [15]. To address this mismatch, transdiagnostic intervention approaches and user-centered design processes have been suggested as potential solutions [9, 12, 16]. Transdiagnostic approaches may consist of intervention components that target one common underlying factor (e.g., emotion regulation difficulties) associated with multiple problems, or multiple components or common therapeutic principles tailored to fit multiple types of problems within the scope of a single intervention [9]. User-centered design approaches emphasizes co-creation between intervention developers and users to promote ecological validity and usability of intervention measures [12]. The objective of the present study is to investigate the effectiveness of a novel, co-created, transdiagnostic preventive intervention, “Supportive parents – coping kids” (SPARCK), consisting of both components targeting common underlying factors and common therapeutic principles.

SPARCK is a transdiagnostic manualized parent training intervention for prevention of child mental health problems designed for implementation in different types of municipal services in Norway. The target group includes families with children displaying elevated symptoms of internalizing mental health problems, i.e. anxiety and depressive symptoms, and/or externalizing mental health problems, i.e. conduct, opposition, and disruptive behavior problem symptoms. The aim of SPARCK is to provide a preventive frontline mental health intervention that is personalized to fit the needs of the client families, as well as service requirements. By reaching a large user group in need of intervention and various municipal services, SPARCK is designed to promote effectiveness and equity in access to care for children and families across municipalities of different sizes and resource availability [17]. Specifically, the transdiagnostic attribute of SPARCK should be particularly relevant for numerous small municipalities in Norway, which often face resource constraints in implementing diverse interventions for various issues.

### Background for the SPARCK content

There are various motivations for developing a parent-based preventive intervention. Most parents spend much time with and are highly involved in the lives of their children. Therefore, they posit a unique position to provide emotional support, promote basic life skills, and make changes in their children`s day to day life – also after an intervention is terminated. Moreover, there is substantial research suggesting that the quality of parent–child interaction is associated with short and long-term outcomes for children’s internalizing [18] and externalizing problems [19]. As such, parents may play a crucial role in preventing and mitigating mental health problems in children [20]. Specifically, a parent-based intervention makes it possible to address modifiable parenting practices that are associated with maintenance of child symptoms and problems. Adding to this, with a parent-based intervention, other potential barriers in child directed interventions can be avoided (e.g., the child is not motivated, the child and/or parent is worried about stigma, avoiding a focus on the child as the problem).

It is well established that harsh and insensitive parenting practices are associated with development and maintenance of externalizing problems [19], which has led to the development of different strands of family-focused interventions for such problems. The first strand is sensitive relationship enhancement, which promotes secure attachment through teaching parents to be sensitive and responsive to the child’s needs, for instance Attachment and Biobehavioural Catch-up (ABC; [21]). Interestingly, research suggests that enhancement of attachment through child-led play is effective not only for decreasing child disruptive behavior [22], but also reducing child depressive symptoms [23]. As such, sensitive relationship enhancement is a good candidate for a parent-based intervention targeting both externalizing and internalizing problems. Second, emotion socialization theory emphasizes how parents may help children with their emotions through emotional coaching [24, 25]. Research suggests that emotion focused interventions are effective for improving child emotional competence [25] and the Tuning Into Kids intervention have been found to prevent and reduce child externalizing behaviors in a Norwegian context [26]. Moreover, the effect on child symptoms has been found to be mediated by pareting practices [27]. Additionally, emotion socialization interventions are also suggested to prevent and reduce internalizing behaviors in children [28, 29]. Third, behavioral management interventions, for example those based on the social interaction learning model (e.g., Generation PMTO; Parent Management Training – Oregon model), are based on the notion that child disruptiveness develops because of parents rewarding negative behaviors rather than positive behaviors [30, 31]. The social interaction learning model highlights how coercive family processes play a crucial role in the development and maintenance of child behavior problems [31]. In such interventions, positive parent–child interactions are promoted by encouraging cooperation, teaching positive behavior, and through consistent and appropriate limit-setting. Behavioral-management interventions are well-established as effective in reducing externalizing probems [32], but research also suggest potential for reducing child internalizing behaviors [33]. However, there is indication that the externalizing parent training programs need to be optimized to better fit the internalizing symptom domains [34]. In sum, these three types of family focused interventions have been found effective in reducing externalizing problems, although through somewhat different pathways. Addtionally, there is research suggesting that the family focused interventions may affect child internalizing behaviors, making each of them suitable for a transdiagnostic intervention.

Attachment- and behavior-based interventions are also suggested to reduce maladaptive and neglectful parenting [32, 35]. Stressful family environments can impact parental and child stress regulation, notably by elevating hypothalamic–pituitary–adrenal (HPA) axis activity, affecting cortisol and DHEA release [36–38]. Dysregulated cortisol levels and suboptimal cortisol-DHEA ratios have been linked to brain development disruption, cognitive issues, and subsequent internalizing and externalizing mental health problems [39, 40]. Some studies suggest that interventions targeting maladaptive parenting improve caregiver-reported stress, atypical child cortisol levels, and child mental health [41, 42]. Yet, meta-analyses of parenting intervention effects on cortisol have revealed limited impact probably due to methodological limitations (e.g., small samples) and potential ineffectiveness in altering parenting processes [43]. Further, a key issue is that daily salivary cortisol measurements, which is often used, can be unreliable indicators of chronic stress due to diurnal and acute stress influences [43]. A relatively new and promising measure of chronic stress is hair samples, as it measures HPA axis activity over a longer period [44] and thus may represent a more robust marker of chronic stress. Using hair samples may yield effects of parenting interventions on stress hormones and child mental health as theoretically assumed. One objective of the present project is to study if SPARCK is effective in affecting stress hormone regulation as indexed by levels of cortisol and DHEA in hair samples.

Finally, recent research proposes parents as change agents in addressing child internalizing symptoms through alternative approaches beyond those designed for externalizing issues [45–48]. Parent-led cognitive behavioral therapy (CBT), in which parents are empowered to implement strategies in their child's daily life, has been found to be effective [45, 49]. Furthermore, a new research area underscores the substantial role of parental accommodation in perpetuating anxiety symptoms [50, 51]. Family accommodation refers to parents’ behaviors to help the child avoid feelings of distress and anxiety. Avoidant and excessive parental support may reinforce children’s problems and contribute to maintenance or worsening of the symptoms. Initially identified in obsessive–compulsive disorder (OCD), family accommodation now extends to anxiety disorders [52]. Despite positive intentions, such parental responses and avoidance behaviors can inadvertently reinforce children's problems. Moreover, it has been suggested that family accommodation is associated with depressive symptoms, for instance by aiding social withdrawal and engaging in unproductive problem discussions [52] and co-rumination, which refers to excessive discussions with the child about his or her thoughts and problems [53].

Summing up, despite support for some parent directed interventions on child internalizing problems the evidence is still inconclusive, and more research is warranted. For example, family accommodation has been proposed as a key component in family-focused interventions for child anxiety problems [51] but is less studied in prevention samples and in relation to child depressive symptoms. In general, support for preventive parenting interventions has been found for some groups of vulnerable children and families. However, the question of effect of parenting interventions for proximal factors like parenting practices and parental stress, and for more distal, yet important factors like child and youth mental health and stress, has still been pointed out as a current major knowledge gap [54].

### Development and optimization of SPARCK

Inspired by design-based research models focusing on intervention content development such as Multiphase Optimization Strategy, IDEAS impact framework and user-centered design process [15, 55, 56], SPARCK was co-created, tested, and optimized in iterative mixed-methods test-cycles with 31 families and 14 frontline practitioners from 2018 to 2023 [17]. Researchers, clinical psychologists, and service practitioners worked together to develop the theoretical basis, components, and Decision support system accompanying intervention strategies (Fig. 2, see methods section for more detailed description). The Social Interaction Learning model, which forms the basis for the theory of change in PMTO, was used as the starting point for the development of SPARCK. Additionally, the program includes empirically supported components based on attachment theory, emotion socialization, cognitive-behavioral therapy (CBT), and family accommodation. As such, SPARCK addresses problems in families with children displaying externalizing, internalizing, and caregiver challenges, by using intervention components collected from different theories to provide a diverse toolkit to personalize content. The dosage in SPARCK is between 3 and 12 individual sessions, with an optional booster session after three months. SPARCK components are tailored to the needs of the families and children guided by the Decision support system, which is inspired by other transdiagnostic approaches, such as MATCH (Modular Approach to Therapy for Children with Anxiety, Depression, Trauma, or Conduct Problems; [57]. Beyond symptomatology and intervention concepts, SPARCK is essentially about helping parents to promote vital coping skills in their children to deal with daily stressful situations, and to promote healthy child development and parent wellbeing.

### Aims and research questions

We will conduct a randomized controlled trial to investigate the effectiveness of SPARCK compared to usual care (UC) in Norwegian frontline services. In Norway, frontline municipal service sectors, such as child welfare, health, school health and school psychological counselling services, are responsible for providing targeted preventive interventions. The primary objective is to examine whether SPARCK is effective in preventing and reducing negative outcomes and promoting positive outcomes for children and parents. It has been pointed out recently [58], that studies on prevention of anxiety should assess actual disorders in a long-term perspective, and we answer this call by including register data on referrals to specialized services and/or contact with child welfare services. We will also conduct a parallel implementation study to examine the relationship between implementation determinants and clinical and implementation outcomes. However, in this study protocol we focus on the effectiveness part of the project. A separate implementation protocol will be written.

Considering the variability in the quality and quantity of the mental health services for children, youths, and families in the frontline services across Norway, in combination with the theoretical and empirical background presented above, our aim has been to develop a flexible and tailored intervention to address heightened, yet subclinical, symptoms of anxiety, depression, and/or externalizing problems among children and youths. Building on our understanding of the significance of engaging with parents, we posit that alterations in child symptoms will, in part, hinge on corresponding changes in parenting practices. We assume that enhancing parenting practices aimed at fostering children's proficiency in essential life skills could exert a positive influence on both healthy child development and parental well-being (see Fig. 2). Consequently, such interventions may serve as a preventive measure against more severe or enduring mental health problems.

We posit several hypotheses to guide the investigation of SPARCK. More specifically, our primary hypothesis is: H1: Children of parents receiving SPARCK will exhibit lower levels of internalizing and/or externalizing symptoms compared to those who receive UC at a) the end of the intervention and b) 6 months post-intervention. Our secondary hypotheses are: H2: Parents in the SPARCK condition will demonstrate better parenting practices, self-efficacy, and less parental stress than parents in the UC condition at a) the end of intervention and b) 6 months post-intervention; and H3: The effect of SPARCK on child symptoms is mediated by changes in parenting practices; H4: Children (≥ 7 years) and parents in the SPARCK condition will report better subjective quality of life than children (≥ 7 years) and parents in the UC condition; H5: Among those who display abnormal blunted or elevated stress hormone levels as indicated by cortisol and DHEA at pre-intervention, children and parents in SPARCK will be more likely to change towards normative levels at post-intervention than those who receive UC; H6: Children in the SPARCK condition will display lower levels of school refusal than children in the UC condition at a) the end of intervention and b) 6 months post-interventions; and H7: Children in the SPARCK condition will be less likely to be referred to child and adolescent outpatient clinics (Norwegian abbreviation: BUP) or have contact with the Child Welfare Services (CWS) in the following two years compared to children in the UC condition.

## Methods

### Study design

This study is a two-armed parallel group randomized controlled effectiveness superiority trial with assessment of key variables at pre-, post-, and six-months follow-up. Participants (i.e., parents) will be allocated either to receive SPARCK or UC.

### Participants, recruitment, and randomization

The target group in this study is caretakers of children between 4 and 12 years who display elevated symptoms of internalizing and/or externalizing problems, but who have not been formally diagnosed or who are not currently undergoing examination in outpatient clinics for a potential diagnosis. The children and/or their caretakers are referred to or seek help from municipal level frontline services. The study is conducted in 24 Norwegian municipalities that vary in size and demography, and together represent all health regions in Norway. All participating municipalities are part of a municipal implementation network hosted by Norwegian Center for Child Behavioral Development (NUBU; Norwegian acronym), and thus have experience with implementation of EBIs in their services. The 24 data collection municipalities have signed a written agreement and committed to contribute with at least two practitioners to be trained in SPARCK, whom each will deliver four intervention cases during the data collection period. Additionally, all municipalities will provide one case to the UC condition for each case to the SPARCK condition. Both SPARCK practitioners and practitioners in the control condition work in municipal frontline services. SPARCK practitioners will not provide cases to the UC condition.

**Fig. 1:**
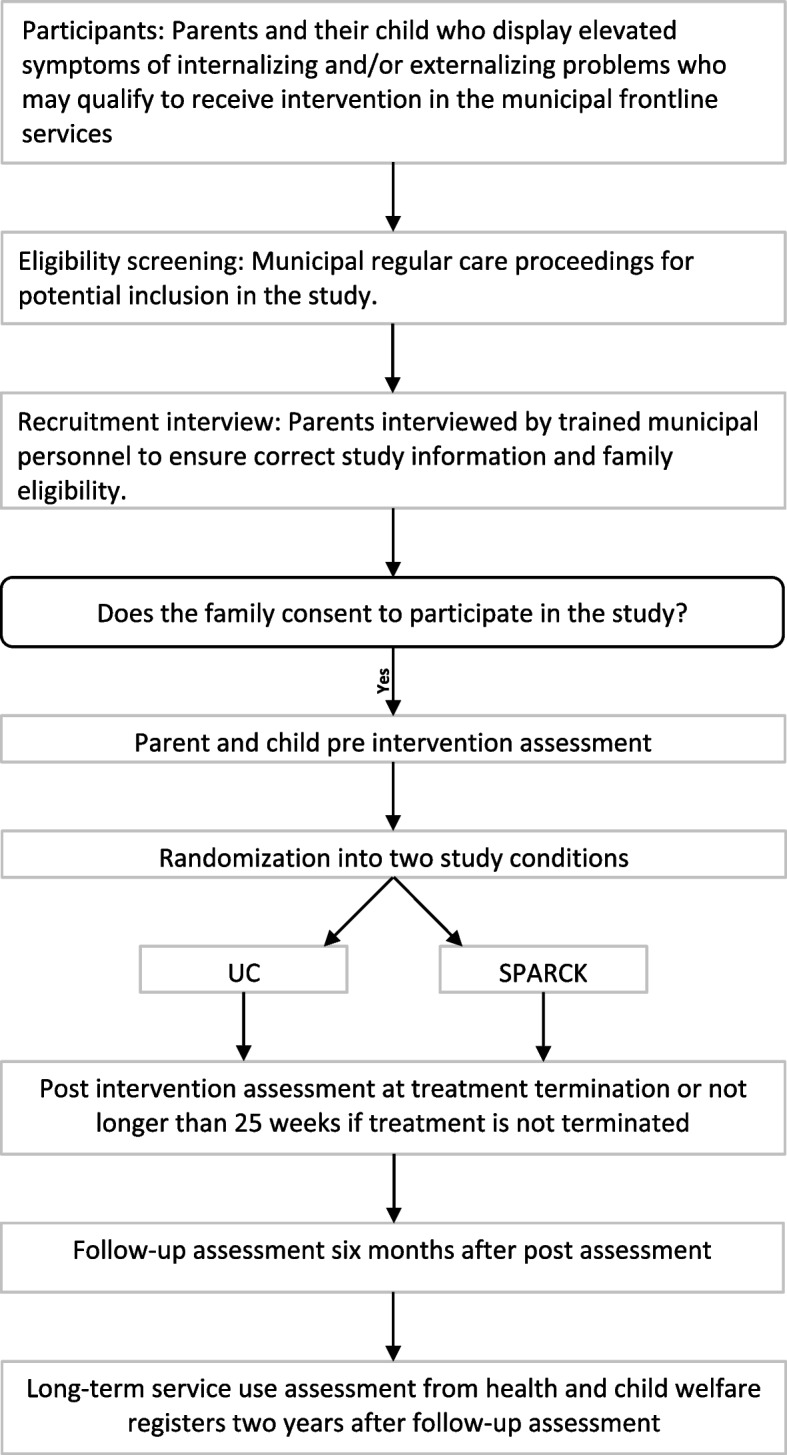
Flow of participants in the study

Participant recruitment will follow regular care procedures for screening and inclusion into frontline services. Practitioners will use their clinical judgments to infer whether participants display problems compatible with the target group in the study (see eligibility criteria). No formal standardized assessment procedures for inclusion in the project will be implemented. However, some municipalities have already implemented more formal regular practice procedures for screening and will continue to utilize these in this study. After the family has been identified as a possible case for the study, a practitioner will conduct a recruitment interview to inform about the study and to ensure that the family is eligible according to inclusion and exclusion criteria (see eligibility criteria below). Written informed consent will be obtained from eligible families before inclusion in the study, see Fig. 1.

Randomization will be performed using an online database hosted by the external provider, Klinforsk, at the Norwegian University of Science and Technology [59] www.klinforsk.no. Accordingly, the allocation sequence will be blinded for researchers, study personnel and practitioners working in the municipalities. Stratified pairwise randomization will be used, meaning that pairs of children/familieswill be recruited from each municipality after which one child/family will be randomized to receive SPARCK and the other to UC. If a second case is not recruited within a four-week period, a single case block randomization backup solution will be used to prevent prolonged delays for the first recruited child/family. In case of such single case randomization, the block size will be blinded for researchers, study personnel and practitioners in the municipalities, to prevent bias in the recruitment process. Participants will be randomized after signing a written informed consent and completing the pre-assessment.

While blinding is difficult or even unfeasible in human-delivered interventions like SPARCK and in the UC condition interventions, efforts have been made to mask participants from knowing which study condition they belong to. All information materials use neutral language such as "condition 1" (UC) and "condition 2" (SPARCK), and municipal recruitment personnel are trained not to provide any biased information to participants. However, it may be challenging to maintain masking during and after receiving the intervention, and participants may figure out which study condition they belong to if practitioners reveal the study condition.

### Eligibility criteria

The target group of this study is children aged four to 12 years who display symptoms of internalizing and/or externalizing problems, and their parents. Participants are excluded if; (a) the child is referred to, or receives, treatment in the specialist services for child and adolescent mental health (BUP) for problems within the internalizing or externalizing domains, (b) the child is diagnosed with pervasive developmental disorder, post-traumatic stress disorder or psychosis, (c) the child exhibit signs of acute risk for suicide, (d) there is documented or suspected ongoing physical or sexual abuse, or (e) the child or the parents receive other systematic interventions targeting the inclusion criteria at the time of recruitment and during the study period. All participating municipalities have implemented PMTO and the PMTO short-form Brief Parent Training for externalizing problems. Since SPARCK partly includes PMTO-based components, neither PMTO-based interventions can be provided as condition 1 intervention, as we would run the risk of evaluating similar content in both conditions for a large proportion of participating families.

### Retention and project implementation

To ensure successful implementation of the study, the host organization (NUBU) collaborates on data collection with local study coordinators in each participating municipality. The study coordinators at the host organization *oversee* the recruitment and data collection process at all the 24 sites. This includes activities concerning the externally provided randomization module, distribution and collection of research data, and to provide support to local study coordinators in the municipalities. The local study coordinators handle the practicalities in the participant recruitment, administers randomization results, organizes practitioners, monitors intervention start-up and end dates in both conditions, monitors data collection, and provides relevant study information to municipal frontline services. The host organization researchers and clinical personnel are not directly involved in the data collection. However, a NUBU core group of researchers and clinical personnel will assist the frontline services when they need research study assistance. For example, problem solving, consideration of eligibility criteria in specific cases, when to discontinue intervention in cases where symptoms are worsening, and to provide information to leaders. Furthermore, the core group will conduct ongoing audits of the trial based on information provided by the collaborating sites and SPARCK supervisors.

To further mitigate the risk of potential harm resulting from participants receiving SPARCK in the study, we have implemented several measures. Firstly, we will conduct an interim data inspection halfway through data collection period to evaluate the quality of the data collected thus far. A data monitoring committee will be supervised by an external researcher who is not involved in the project, and who will oversee the data inspection process and make any necessary decisions. In addition, during the interim data inspection, we will evaluate the balance of the study design in terms of internalizing and externalizing symptoms. If the data reveals any imbalance, we will review the research questions and hypotheses accordingly, to ensure that our analysis is appropriately informed by the data collected.

### Intervention conditions

#### Condition 1: Usual care

The UC condition refers to the standard clinical practice in the Norwegian frontline services, which varies in scope and intensity across different municipalities. In total, the 24 municipal sites have listed 23 manualized interventions that are planned to be used as UC, in addition to un-structured counselling. Eleven out of the 23 manualized interventions can be deemed evidence-based, supported by empirical documentation of intervention effectiveness. The median number of potential manualized UC interventions per municipality is three. Two municipalities have only listed un-structured counselling with professional practitioners as UC, whereas 23 have listed unstructured counselling in addition to manualized interventions. In line with the heterogenic Norwegian frontline service system, the practitioners in both the UC and SPARCK condition will vary in background training and clinical competence. Professions listed by the 24 municipalities include public health nurse, social worker, psychologist, educational psychological counsellor, and physician.

In the UC condition, intervention can be given to the parents, to the child or to a combination of parents and child – either together in sessions or sequential. There is no minimum or maximum number of sessions or weeks in the UC condition.

#### Condition 2: SPARCK intervention

**Fig. 2:**
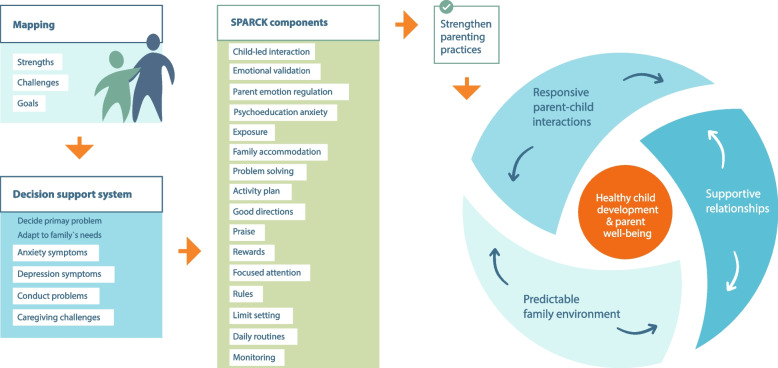
Conceptual model of the SPARCK theory of change

SPARCK is a transdiagnostic preventive intervention designed to target both internalizing and externalizing problems. The primary objective is to enhance developmentally appropriate parenting practices that may foster sensitive parent–child relationships and nurture the fundamental core skills necessary for healthy child development. A conceptual model of the SPARCK theory of change (ToC) is displayed in Fig. 2.

SPARCK is designed to be delivered in up to 12 weekly 1-h individual face-to-face sessions, ideally attended by both parents. If deemed necessary, the practitioner and family can decide together to adjust this plan, for instance to have longer intervals between sessions (excluding the introductory sessions), or to have online sessions. The first step in the SPARCK intervention, the Mapping component, is fundamental for the customization of intervention components to the families’ needs. During this session, parents collaborate with practitioners to make clear and concise goals in intervention (see Fig. 2). Importantly, the goals (maximum three) must include child and/or parent behavior and/or parent–child interactions that are amenable to change (e.g., *Child goes to sleep in own bed; We as parents stay calm when helping with homework)*. The parents` goals, together with information from any referral papers and assessments carried out prior to and during the Mapping session, are used to get a picture of the strengths and challenges experienced by the families and to choose what appears to be the primary problem that should be addressed first.

The various components outlined in Fig. 2 include a broad range of intervention strategies for parents to use in their daily interactions with their children. The components typically begin with sharing knowledge, for instance on the importance of emotional validation for emotion regulation, the rationale for exposure, and the value of predictable routines for child well-being. Further, the components outline examples of helpful questions to engage the families and make the topic relevant. Additionally, all components include role plays to model and practice specific skills, such as how to validate the child`s emotion, give directions, or ways to talk with the child about exposure. A central part of many components is a description of how to develop a step-by-step plan tailored to each family`s specific goals. All components also include examples of homework assignments. Essentially, SPARCK can be conceptualized as a parent training intervention by targeting child symptoms via change in parent–child interactions and parenting behaviors. However, SPARCK also incorporates components that can be seen as parent-led CBT, such as the parent-led Exposure component.

To help the SPARCK practitioner make the choices of components that may fit the family’s needs, a Decision support system (DSS) consisting of four recommended tracks outlines which components to use and in what order. The recommended tracks target 1) anxiety symptoms, 2) depression symptoms, 3) conduct problems, and 4) caregiving challenges. For example, the DSS recommended track for addressing anxiety issues involves Mapping, Emotional validation, Psychoeducation anxiety, and Exposure and/or Family accommodation. Additionally, the DSS provides guidance on when to incorporate other components. Even if the family reports multiple concerns, a recommended track is still to be chosen to ensure a clear focus. To allow for this flexibility, SPARCK components are designed as stand-alone components, allowing practitioners to choose and combine them throughout the course of intervention. However, there is a sequential nature of the components, advising that the SPARCK practitioners should adhere to a prescribed order. For example, Emotional validation and Psychoeducation anxiety should be addressed before parental supported Exposure, while Good directions and Rewards before Limit setting. Parents are given the option of bringing the child to a session at the beginning of the course of intervention to provide the child with information about SPARCK, as well as the opportunity to share any wishes for change. If considered necessary, the parents may also bring the child to a subsequent session (e.g., for psychoeducation, brainstorming or to practice specific skills). Any inclusion of the child is always conducted jointly with their parents.

The SPARCK practitioners work in the municipal frontline services and thus have comparable training and educational background to the practitioners in the control condition. However, all SPARCK practitioners have previously undergone training in PMTO. The SPARCK training of practitioners consists of a four-day training program that familiarize the practitioners with the content of SPARCK. Furthermore, the training program includes a weekly one-hour supervision session for pairs of SPARCK practitioners during their first two cases. For subsequent cases, supervision dosage is reduced to one hour every second week for the pairs of SPARCK practitioners. The initial weekly supervision is part of the SPARCK training and enables a more practical form of learning the content, that is, through discussion of issues that arise along the course of sessions with the parents. The SPARCK practitioners will receive supervision from specialists in clinical psychologists from NUBU or from members of the host organizations’ National Implementation Team, all of which have undergone previous training in and practice with SPARCK.

### Data collection

In this study we use a combination of web-based questionnaires to be completed by one parent on mobile phones, pen-and-pencil questionnaires to the children for those who are seven years or older, and hair samples from one parent (i.e., the parent who answers the questionnaires) and the child to measure biomarkers of stress (i.e., cortisol and DHEA). The families receive a hair-sample kit with instructions and necessary equipment for cutting the correct amount of hair. The pen-and-pencil questionnaires for the children is distributed together with the hair-sample kits.

Parent reported data (i.e., the web-based questionnaires by one parent) will be gathered at three time points; pre (T1), post (treatment termination, T2), and six months follow-up (six months after post, T3; see Fig. 1). If the UC group receives open-ended treatments or counselling is delayed in either condition, we will gather T2 parent reported data at 25 weeks after T1 (i.e., T2 is gathered 25 weeks after T1 at the latest). Hair samples will be gathered through participant self-sampling from one parent and the child in all families. Both the hair samples and the child report pen-and-pencil questionnaire is gathered in connection with T1 and at T2. They are instructed to send a text message to the NUBU study coordinators when they have posted the hair-samples and the child questionnaire in the mail, upon which they are ready to be randomized. At T2, the hair-sample kit and the child questionnaire are sent out approximately four weeks after the termination of the intervention (or two weeks after week 25). The delay in the data gathering of hair-samples is simply because the hair-samples reflect the retrospective average bio-marker levels across an 8-week period (two-centimeter hair segment samples).

Finally, register data from Statistics Norway for CWS service contacts and from the Norwegian Patient Register for service referrals will be collected. Data will be obtained two years after post-treatment. Parents have consented to collection of register-data up to 5 years after follow-up, but the potential five-year long-term register data collection depends on the project receiving additional funding.

### Measures

Various measurement instruments are used to investigate the primary hypothesis of parent reported change in child externalizing and internalizing symptoms. and the secondary hypotheses testing various outcomes such as change in proximal family relational outcomes, and other more distal child and parent level outcomes, including moderators, mediators and background factors. An overview of assessment instruments is displayed in Table 1.

**Table 1:** Measurement instruments

Measures	Respondent	Level outcome	T1	T2	T3	T4	Weekly assessed
ECBI	Parent	Child	x	x	x		
RCADS-P	Parent	Child	x	x	x		
SDQ-P	Parent	Child	x	x	x		
SRQ	Parent	Child	x	x	x		
Kid-KINDL	Child	Child	x	x^1^			
PAFAS	Parent	Relational	x	x	x		
FRQ	Parent	Relational	x	x	x		
PAB	Parent	Relational	x	x	x		
MaaP-SF	Parent	Parent	x	x	x		
PSS	Parent	Parent	x	x	x		
SWLS	Parent	Parent	x	x	x		
HSCL-10	Parent	Parent	x	x	x		
LES	Parent	Parent	x	x	x		
BioM	Child	Child	x	x^1^			
BioM	Parent	Parent	x	x^1^			
PRQ	Practitioner	Service & Intervention	x	x			
FidQ	Practitioner	Intervention					x
WAI-S	Practitioner	Intervention		x			
WAI-S	Parent	Intervention		x			
UserSQ	Parent	Intervention		x			
RBD	Registers	Child				x	

The parent questionnaires take approximately 20 min to complete. Parents receive 200 NOK (approximately 20 USD) as compensation for the time spent completing the questionnaires and providing hair samples. Instruments with positively formulated items are strategically interspersed among the instruments with negatively formulated items.

For the primary outcome on externalizing problems at T1-3, we will use the parent reported Eyberg Child Behavior Inventory intensity scale (ECBI; [60]) which assesses frequency of children’s externalizing symptoms. For the primary outcomes on internalizing symptoms, we will use the Revised Child Anxiety and Depression Scale parent version (RCADS-P; [61]). In addition, as this is a community level sample with heightened, but not clinical level, symptoms, and may include both children with externalizing and/or internalizing problems, we include the Strengths and Difficulties Questionnaire parent report (SDQ-P). The 20-item total problem scale from the SDQ-P, which assesses multiple correlates of internalizing and externalizing symptoms in children, is included as a primary outcome [62].

Secondary child level outcomes will be assessed utilizing the following instruments at T1-3: For the parent reported child behaviors we use a school refusal questionnaire (SRQ) that has been constructed for the SPARCK project to tap parent reported child school refusal behaviors. The items are reported on a 5-point scales ranging from “Very rare” to “very often”. For example, “In the last month, how often have your child said that he/she doesn’t want to go to school”. For child self-report of subjective quality of life, children aged ≥ 7 years (or who turn seven within that specific year) will fill out the 24-item Kid-KINDL [63] on paper-and-pencil version assessed at T1 and at four weeks past T2.

Secondary parent reported family outcomes at T1-3: The Parenting and Family Adjustment Scales (PAFAS) is used to assess parenting practices and family interactions and relationships [64]. A 7-item family routines questionnaire (FRQ) was purposefully developed for the current trial. This instrument aims to gauge both the frequency and extent of cooperation within everyday routine scenarios. The questionnaire comprises three items focused on the frequency of daily routines, employing a 5-point Likert scale that spans from "completely disagree" to "completely agree." For instance, “My child repeats the same procedures every night before going to bed”, and “My child and I share one meal at least once daily”. Additionally, the family routine questionnaire encompasses four items designed to evaluate the degree of cooperation prevalent in daily routines, assessed on 7-point scales ranging from “Very bad” to “Very good”. A questionnaire on family accommodation has been systematically developed to assess parental accommodation behaviors (PAB) in response to child difficulties. The questionnaire comprises 14 items, encompassing distinct dimensions of accommodation. Among these, five items pertain to parental accommodation behaviors within the context of daily family routines and four items examine parental accommodation within the realm of the parents' own life spheres, including work, social interactions, leisure activities, and sleep patterns. Moreover, the questionnaire incorporates five items targeting parental avoidant behaviors. For instance, respondents are asked to indicate the extent to which they permit their child to abstain from participating in activities commonly undertaken by children of comparable age.

For parent-level outcomes, several parent-reported instruments are assessed across T1-3. Me as a Parent, short-form (MaaP-SF; [65]) measures parental self-efficacy. The Perceived Stress Scale (PSS), consisting of 10 items, evaluates parents' subjective perception of stress [66]. Parents global perception of life quality is assessed with the Satisfaction With Life Scale (SWLS; [67]). Psychological distress is assessed using the 10-item version of the Hopkins Symptom Checklist (HSCL-10; [68]). Parents exposure to positive and negative life events in the last year is captured with the Life Events Scale (LES; [69]). Various background factors such as demographics and social and economic resources, family constellation etc., will be reported by parents and obtained at T1 and T3 assessments.

Biological markers of stress hormones levels (BioM), cortisol and dehydroepiandrosterone (DHEA), will be collected from 2 cm scalp hair samples collected at T1 and four weeks after the parent web-based T2 assessments, as 2 cm hair scalp samples contain approximately 8 weeks of biological material. The target child and the primary caretaker provide the hair samples. If the intervention is delayed in the study conditions, hair samples will be collected 4 weeks after week 25. Hair samples are self-sampled, wherein parents are provided with hair sampling kits encompassing all the required materials and comprehensive instructions elucidating the process of segmenting and cutting the hair samples. If parents face difficulties with the collection of hair samples, they are instructed to seek assistance or contact NUBU study coordinators.

In both study conditions we digitally assess intervention content, practitioner characteristics, and service-related details as reported by practitioners. For practitioners in the UC condition 1, a practitioner report questionnaire (PRQ) has been developed to evaluate therapist background, service-related aspects, educational background, training in EBIs, and counselling experience etc. Moreover, PRQ at T2 evaluates whether an EBI were provided and what intervention components that were given to families in the condition 1 counselling. Parallel assessments are conducted for the condition 2 SPARCK practitioners on practitioner and service characteristics. Additionally, condition 1 practitioners supply information regarding intervention administered to condition 1 families, outlining if a standardized or EBI has been provided, intervention components, session frequency, dosage, recipients, and targeted issues. Condition 1 practitioners provide their background and service-related details at intervention onset, whereas intervention specifics are reported upon completion or by week 25. In contrast, condition 2 practitioners complete a web-based fidelity questionnaire (FidQ) designed and optimized during the SPARCK development phases, detailing recipients, intervention strategies employed, pedagogical tools used, client engagement, and parental assessment of Goals in intervention. This SPARCK questionnaire is completed weekly after each session. Condition 2 practitioners report service and practitioner characteristics at project start-up. Parent and practitioner reported working alliance will be assessed using the 12-item Working Alliance Inventory – short form (WAI-S; [70]). Parent reported satisfaction with intervention will be reported with a User satisfaction questionnaire (UserSQ) adapted from the Family satisfaction survey [71]. UserSQ contains 5-items assessed on 5-point Likert scales ranging between “Not correct” and “Correct all the time”. Finally, register outcomes on child referrals to mental health outpatient specialized services and CWS services (RBD) will be collected two years after T3 follow-up assessments.

### Data analyses

The effect of the SPARCK vs UC will be indicated through a group (between) by time (within) interaction effect in a mixed effect repeated measures design. Respondents will be included in the analysis if they are assessed at T1 and randomized following the intention-to-treat principle. Missing data at any timepoint will be handled with appropriate missing data techniques depending on the type of data and missing data patterns. For the parent and practitioner-reported web-based questionnaires, completion of all instrument items is mandatory, ensuring that there will be no missing data at the item level. With an expected weak effect size of f = 0.1, GPOWER 3.1 estimates the necessary n to detect group by time interaction with 80% power to be 164, but this is based on no design effects and no dropout. Assuming a therapist intraclass correlation of 0.08 [72], with 4 cases per therapist, the design effect is 1.24, giving an effective n of 80% of the nominal n. Correcting for design effects and dropout the needed effective sample size is 252. Further, a dropout of 20% can be assumed. Forty-nine potential SPARCK practitioners across 24 municipalities have already committed to contribute. With four cases per SPARCK practitioner and four control cases, the total potential N is currently 392. We are thus within reach of a planned 20% safety margin to account for practitioner and participant drop-out.

### Data management

To ensure the security and privacy of sensitive information, we use the University of Oslo's Services for Sensitive Data (TSD) for all data storage and analysis. We use Nettskjema, also developed and operated by the University Information Technology Center, a TSD-integrated survey solution for collecting sensitive data. All study information, including participant identification keys and raw data, will be securely stored in TSD. Access to this information will be restricted to authorized personnel. Specifically, only study coordinators will have access to participant identification keys and raw data, while researchers will only have access to de-identified data. We will also implement measures to protect data confidentiality and integrity, including encryption of all data transfers and regular backups to safeguard against data loss. In the event of any breaches or unauthorized access, we will follow established protocols for reporting and responding to such incidents. Hair samples and paper questionnaires will be adequately secured and stored at NUBUs’ locations.

## Discussion

The SPARCK intervention is a novel intervention that requires empirical evidence about effectiveness in a randomized trial. The current study has the potential to make a significant contribution in this regard and to the understanding of how a preventive transdiagnostic intervention may perform in Norwegian regular care services.

The primary objective in this study is to test the effect of SPARCK when compared with standard care. Accordingly, the current study may shed light on how a transdiagnostic preventive intervention can yield favorable outcomes or not when compared to focal interventions in standard care typically more focal in scope aimed at delimited target groups. A related question is whether the inherent transdiagnostic and personalized feature of SPARCK may also enhance effectiveness by allowing for flexible tailoring of content to fit the specific needs of each family. If SPARCK yields treatment effects over and above standard care, SPARCK holds the potential to innovate the municipal frontline services with a flexible and effective parenting intervention that may reach a large user group in need of frontline support services. At the service level, the transdiagnostic feature of SPARCK is customized to a to a varied landscape of Norwegian municipal frontline services, which differ in size, resources, and organizational structure. In particular, municipalities in small and rural areas may lack resources that is required to implement multiple evidence-based interventions (EBIs) for specific problems. If proven effective, SPARCK can promote the frontline usability and delivery of effective intervention content to larger segments of end users, thereby supporting equity in care throughout Norway's heterogeneous municipal landscape.

The heterogeneity in end-users, services, and municipalities reflect the regular practice realities [73]. Currently, we have chosen to conduct an effectiveness study in a naturalistic setting, and not in a highly controlled efficacy-study, as performed in the SPARCK optimization cycles. However, this choice brings along some limitations regarding heterogeneity of child symptomatology when analyzing outcomes. This is a particularly relevant problem when analyzing outcomes from transdiagnostic interventions. For example, if children do not display comorbid internalizing and externalizing symptoms, the non-present symptom may statistically mask the effect on the symptom which is present. For example, if a child displays anxiety symptoms only, it is expected to observe a change primarily on anxiety symptoms but not, or only to a small degree, on conduct and depression symptoms. However, being a transdiagnostic intervention, the outcome is nevertheless measured as consisting of all three symptom domains. Thus, the heterogeneity in the sample may lead to an underestimation of treatment effects. In this regard, the design per se is not put up to explain for instance for which group of main symptoms SPARCK is better (or effective). Other and more general limitations will also apply in RCT, for instance which of the SPARCK components are effective for what groups or if SPARCK is effective in some services, but not others. However, mediation and moderation analyses in addition to the implementation part of the study (not described here) will hopefully contribute to shed light on such questions.

Another systematic difference between study conditions worth noting is the background training of the practitioners. For example, we use trained PMTO therapists to deliver the SPARCK condition. The PMTO therapist have previously undergone a 21-day training program including training in active teaching skills such as role play and problem solving, and clinical process skills. Thus, the SPARCK practitioners all possess some content and process skills that may have enabled them to learn the SPARCK material competently. On the other hand, practitioners in the UC condition may possess background training and process skills comparable with PMTO therapists, and the list of interventions supplied by the municipalities describing the potential UC interventions show that many UC practitioners indeed have undergone training in manualized interventions and EBIs. To monitor such potential differences between study conditions, we assess various practitioner backgrounds such as education, years with a relevant job, if they are specialists, training in any EBIs etc. in addition to counsellor alliance and the content delivered in UC.

Relatedly, another potential effect of conducting a randomized controlled effectiveness trial in naturalistic settings is that those who deliver the UC condition provide more and/or better interventions because of being observed in the study. Thus, the municipalities may have mobilized to provide “good enough” UC, which may also bias the results. In the SPARCK RCT design, we have less control over such factors.

We recognize that implementing a large-scale randomized study in regular care is resource demanding and often difficult, and that there are many potential pitfalls that may affect the risk of bias and in turn the knowledge base for which to conclude about effects. Thus, it has been pointed out that future research on family-based interventions should use robust designs, but also be based on a close cooperation between the scientific and the local environments. We answer this call for research anchored in both science, practice, and local communities. With a previously co-created and optimized intervention, we hope that this randomized effectiveness study, together with the implementation study, will contribute with findings that address the challenges related to effectiveness and successful implementation of parenting interventions for prevention of childhood mental health problems.

### Supplementary Information

Additional file1 (DOC 141 KB)

## Data Availability

The datasets generated and/or analyzed during the current study are not publicly available due to General Data Protection Regulation (GDPR) and Norwegian health care research regulations but can be available from the corresponding author in a reasonable request.

## Abbreviations

SPARCK: Supportive Parents – Coping Kids
UC: Usual care
EBI: Evidence-based intervention
PMTO: Parent Management Training – Oregon model
HPA: Hypothalamic-pituitary-adrenal
DHEA: Dehydroepiandrosterone
CBT: Cognitive behavioral therapy
OCD: Obsessive-compulsive disorder
IDEAS: Innovate develop evaluate adapt scale
MATCH: Modular Approach to Therapy for Children with Anxiety, Depression, Trauma, or Conduct Problems
BUP: Norwegian child and adolescent outpatient clinics
CWS: Child welfare services
NUBU: Norwegian Center for Child Behavioral Development
DSS: Decision support system
T1: Time,1 pretreatment
T2: Time 2, posttreatment
T3: Time 3, follow-up treatment
ECBI: Eyberg Child Behavior Inventory
RCADS-P: Revised child anxiety and depression scale – parent version
SDQ-P: Strengths and difficulties questionnaire – parent report
SRQ: School refusal questionnaire
PAFAS: The Parenting and Family Adjustment Scales
FRQ: Family routines questionnaire
PAB: Parental accommodation behaviors
MaaP-SF: Me as a Parent, short-form
PSS: Perceived Stress Scale
SWLS: Satisfaction With Life Scale
HSCL-10: Hopkins Symptom Checklist
LES: Life Events Scale
BioM: Biological marker
PRQ: Practitioner report questionnaire
FidQ: Fidelity Questionnaire
Wai-S: Working Alliance Inventory – short form
UserSQ: User satisfaction questionnaire
RBD: Register based data
TSD: University of Oslo's Services for Sensitive Data
REK: Regional Committee for Medical Research Ethics in Norway
SIKT: Norwegian Agency for Shared Services in Education and Research
GDPR: General Data Protection Regulation
